# Development of Functional Sourdough Bread Using *Lactobacillus sakei* and Germinated Brown Rice: Evaluation of Phenolic Compounds, Antioxidant Capacity, Gamma‐Amino Butyric Acid (GABA) Content, and Sensory Characteristics

**DOI:** 10.1002/fsn3.70047

**Published:** 2025-02-24

**Authors:** Seyed Ali Sobhanian, Solmaz Saremnezhad, Mostafa Soltani

**Affiliations:** ^1^ Department of Pharma‐Economy, Faculty of Pharmacy, Tehran Medical Sciences Islamic Azad University Tehran Iran; ^2^ Department of Food Sciences and Technology, Faculty of Pharmacy, Tehran Medical Sciences Islamic Azad University Tehran Iran; ^3^ Nutrition and Food Sciences Research Center, Tehran Medical Sciences Islamic Azad University Tehran Iran

**Keywords:** brown rice, functional sourdough bread, GABA, *Lactobacillus sakei*, phenolic compounds, sensory characteristics

## Abstract

The aim of this study was to evaluate the effect of using sourdough containing *Lactobacillus sakei* ( *L. sakei* ) and germinated brown rice on chemical composition, functional characteristics, and sensory properties of leavened bread. In this context, three types of germinated brown rice sourdoughs containing *L. sakei* , *Lactobacillus sanfranciscensis* ( *L. sanfranciscensis* ) and the mixture of *L. sakei* + *L. sanfranciscensis* were prepared and used for bread production. According to the results, a significant decrease in the pH and protein values and a significant increase in the moisture and free amino acid contents were observed in the breads prepared using different sourdoughs compared to control bread (*p* ≤ 0.05). The use of sourdough containing *L. sakei* resulted in a bread with the highest amounts of bound and total phenolic compounds and antioxidant activity. The results also indicated a significant increase in gamma‐amino butyric acid (GABA) content in breads prepared by *L. sakei* + *L. sanfranciscensis* and *L. sakei* fermented sourdoughs, respectively. The *L. sakei* + *L. sanfranciscensis* and *L. sanfranciscensis* containing sourdough breads gained the highest overall acceptability from sensory panelists point of view (*p* > 0.05). Overall, *L. sakei* showed a remarkable potential for use in the production of functional breads.

## Graphical abstract

- Germinated brown rice sourdoughs fermented with different LAB were used in bread‐making.
- The highest antioxidant capacity was seen in *L. sakei* fermented sourdough bread.
- Sourdough fermentation caused an increase in gamma‐amino butyric acid content in the bread samples.

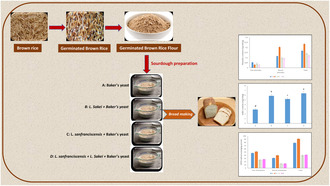

## 1 Introduction

Considering the remarkable demand for food products with enhanced functional values, the development of cereal‐based products with high levels of functionality and acceptable sensory characteristics has received noticeable scientific interest (Kotsiou et al., Kotsiou et al. 2022). Bread, as one of the major sources of carbohydrate intake for the human body, is a common food across the world and can be targeted as an appropriate delivery medium for functional compounds (Amoah et al. 2020; Liguori et al. 2020). During the bread preparation process, sourdough fermentation by lactic acid bacteria and yeast results in improvements in volume, structure, flavor, and shelf life of the final product (Yildirim and Arici 2019). The typical sourdough lactic acid bacteria is *Lactobacillus sanfranciscensis* , with competitive properties and a remarkable effect on the overall quality of bread (Suo, Chen, and Wang 2021; Boudaoud et al. 2021).

Brown rice consists of the endosperm, embryo, and bran of the rice seed and is distinguished as a good source of bioactive compounds, that is, gamma‐aminobutyric acid (GABA), antioxidants, and other phytochemicals with considerable health benefits (Kittibunchakul et al. 2021; Wu et al. 2022). Germination is a common method for improving the functional properties and nutritional profile of brown rice through the formation of bioactive compounds, such as GABA, which has nutraceutical properties including a decrease in blood pressure and protection of brain health. In addition, the flour obtained from germinated brown rice has a proper appearance, longer shelf life, sweeter taste, and better texture and cooking properties compared to the one prepared from white rice. From a nutritional point of view, some amino acids like cysteine and aspartic acid can also be formed as a result of protease and amylase activity after the soaking stage of the germination process (Songsamoe, Khunjan, and Matan 2021; Thomas et al. 2023).

On the other hand, lactic acid bacteria, that is, *Lactobacillus delbrueckii* subsp. *bulgaricus*, *Lactobacillus plantarum* , *Lactococcus lactis* , and *Lactobacillus brevis* as probiotic strains with a decisive role in the food industry, are capable of producing GABA and phenolic compounds with antioxidant activity during the fermentation process (Cui et al. 2020). The use of probiotic lactic acid bacteria can provide the possibility for the manufacturing of health‐promoting fermented foods with bioactive properties such as improvement of gut function and the immune system, prevention of intestinal infections, regulation of blood cholesterol, and increasing the bioavailability of nutrients (Kittibunchakul et al. 2021). *Lactobacillus sakei* ( *L. sakei* ) is identified as psychrophilic lactic acid bacteria that have a rod shape morphology (0.6–0.8 μm) and is usually isolated from plant origin foods. *L. sakei* has an inhibitory impact on food‐borne pathogens like *Listeria monocytogenes* and *Staphylococcus aureus* due to the ability to produce bacteriocins such as sakacin P and sakacin Q. Moreover, the capability of *L. sakei* for the production of nutritive and health‐related metabolites such as GABA with beneficial effects on human health has been proven (Carvalho et al. 2018; Yu et al. 2024).

Regarding the scientific reports about the positive effect of germination on the accumulation of GABA in brown rice, along with the potential of *L. sakei* for GABA synthesis, and the lack of knowledge about the effects of using germinated brown rice and *L. sakei* as a novel microorganism in sourdough fermentation on the functional characteristics of sourdough bread, the aim of the present study was to investigate the effect of *L. sakei* and germinated brown rice on GABA content, phenolic compounds, and antioxidant capacity of sourdough bread.

## 2 Materials and Methods

### 2.1 Materials and Chemicals

Commercial wheat flour (Extraction rate 82%) for bread making was provided by Talaee Parand Co. (Tehran, Iran). The chemical properties of the flour were as follows: moisture: 13.5% ± 0.1%, protein: (*N* × 5.70)/dry matter: 12.5% ± 0.15%, fat/dry matter: 1.2% ± 0.03%, ash/dry matter: 0.55% ± 0.04%, wet gluten: 26.2% ± 0.55%.

Sodium stearoyl lactylate (SSL), diacetyl tartaric acid esters of mono‐ and diglycerides (DATEM), and lecithin were purchased from Pars Behboud Asia (Mashhad, Iran) and Dor Chimi Marjan (Tehran, Iran) respectively. The salt, sugar, sunflower oil, and instant dry yeast (Golmayeh, Tabriz, Iran) were obtained from a local supermarket. Folin‐Ciocalteau reagent, Gallic acid (GA), and 2,2‐Diphenyl‐1‐picrylhydrazyl (DPPH) were purchased from Sigma–Aldrich (St. Louis, MO, USA). All other chemicals were of analytical or HPLC grade.

### 2.2 Germination of Brown Rice

Brown rice (Astaneh Ashrafieh Cultivar, Gilan, Iran) was soaked in water (at 25°C for 24 h) and the soaking water was changed every 8 h. The seeds were drained at the end of soaking, distributed on plastic trays, and covered by double layers of cotton cloth. The germination took place at 30°C and a relative humidity of 90%–95% for 24 h. Germinated seeds were dried at 50°C to approximately 10% moisture content, then ground and passed through a 40‐mesh sieve and kept in a plastic container at −20°C. The chemical composition of the germinated brown rice flour was as follows: moisture: 4.20% ± 0.1%, protein: 32.00% ± 0.11%, ash: 1.90% ± 0.04%, free phenolic compounds: 0.42 ± 0.02 mg GAE/g, bound phenolic compounds: 0.96 ± 0.01 mg GAE/g, free antioxidant activity: 46% ± 1%, bound antioxidant activity: 14% ± 0.5%, GABA: 10.22 ± 0.01 mg/100 g.

### 2.3 Microorganisms and Culture Conditions

*L. sanfranciscensis* (PTCC 1739) and *L. sakei* (PTCC 1712) were provided from the Persian Type Culture Collection (Tehran, Iran). The strains were routinely cultured in MRS broth (Darmstadt, Germany) at 30°C for 48 h (Rizzello et al. 2008). For inoculum preparation, cells were harvested by centrifugation, washed twice with sterile peptone saline solution (10 g/L peptone and 9 g/L NaCl), and re‐suspended in 10 mL of sterile tap water (Dinardo et al. 2019).

### 2.4 Sourdough Preparation

Three different sourdoughs were prepared with *L. sanfranciscensis* (SD1), *L. sakei* (SD2), or a mixture of *L. sanfranciscensis* and *L. sakei* (SD3). For sourdough production, germinated brown rice flour (100 g), instant dry yeast (4.11 g), and maltose powder (2.5 g) were mixed with tap water. The final cell densities in sourdough samples were approximately 10^5^ cfu/g with the 1:1 ratio of the two lactobacilli strains in SD3. Samples were subjected to fermentation at 40°C in a laboratory incubator (RI 55, Rad Teb Novin, Iran) until reaching pH 4.9 ± 0.1.

### 2.5 Bread Preparation

A mixture of wheat and germinated brown rice flours (in the ratio of 9:1 *w/w*) was used for the preparation of bread samples. The basic dough formulation (on the base of 100 g flour) included: salt (1.5 g), sugar (6.5 g), α‐amylase (0.005 g), ascorbic acid (0.05 g), lecithin (1 g), SSL (0.4 g), DATEM (0.5 g), vegetable oil (6 g), instant dry yeast (2.5 g) and sourdough (9% *w/w* of dough weight). The prepared samples were as follows:

A: control bread sample without using sourdough.

B: bread prepared with sourdough containing *L. sakei*

C: bread prepared with sourdough containing *L. sanfranciscensis*

D: bread prepared with sourdough containing the mixture of *L. sakei* and *L. sanfranciscensis*

The ingredients were kneaded in a spiral dough mixer (SPM 40, Fimak, Turkey) for 5 min at low speed. After resting for 15 min at room temperature, the dough pieces were proofed for 30 min (35°C and 76%–80% relative humidity) and finally baked at 180°C for 25 min. The quality characteristics of breads were evaluated after cooling the samples for 1 h at room temperature.

### 2.6 Chemical Composition

A bread of each treatment was analyzed in triplicate for pH, moisture (oven drying method), and protein (micro‐Kjeldahl method) by the methods described in AOAC (2000).

### 2.7 Functional Properties of Bread Samples

#### 2.7.1 Free and Bound Phenolic Compounds

For the extraction of free and bound phenolic compounds, the method of Zargarchi and Saremnezhad (2019) was applied with slight modifications. Briefly, 3 g of bread sample was extracted (twice) with 30 mL of 80% ethanol solution at room temperature and then centrifuged at 4000 rpm for 5 min. The supernatant was used as the source of free phenolic compounds. To obtain the bound phenolic fraction, the residue of free phenolic extraction was digested with 30 mL of 4 M NaOH at room temperature for 1 h under constant stirring, then centrifuged at 4000 rpm for 5 min, and the supernatant was collected as the source of bound phenolic compounds.

To measure the concentration of free and bound phenolic compounds, 1000 μL of each phenolic fraction was added to the mixture of Folin–ciocalteu reagent (1 mL), sodium carbonate (7.5%, 2 mL), and distilled water (10 mL), stirred for 5 min, and placed in the dark for 1 h. Then, the absorbance of the sample was read at 750 nm in a spectrophotometer (Philler Scientific, SU 600, USA). The results were reported as mg gallic acid equivalent (GAE)/g (Shao et al. 2014).

#### 2.7.2 Antioxidant Capacity

The antioxidant capacity of free and bound phenolic compounds was measured according to the method described by Tian et al. (2021) with some modifications. Briefly, DPPH solution (0.075 Mm, 3.9 mL) was added to 100 μL of the sample and stirred for 1.5 h in a dark place. The absorbance of the samples was measured by spectrophotometer at 517 nm. The antioxidant capacity was calculated according to equation 1.

(1) $$\begin{array}{c} \text{DPPH radical scavenging activity}\;\left(\%\right)\\ =1-\left({\mathrm{Abs}}_{\text{sample}}-{\mathrm{Abs}}_{\text{control}}/{\mathrm{Abs}}_{\text{control}}\right)*100 \end{array}$$

#### 2.7.3 GABA Content

Each bread sample (1 g) was extracted with 25 mL trichloroacetic acid (1 h, room temperature) and centrifuged (5590 × g, 10 min). The supernatant (400 μL) was filtered (0.22 μm) and 20 μL of filtrate was analyzed with HPLC according to the procedure described by Al‐Ansi et al. (2020).

#### 2.7.4 Amino Acid Composition

For determining the amino acid composition, 1 g of the bread samples was digested in 10 mL of 6 mol/L HCl (at 110°C for 22 h) under a nitrogen atmosphere. After dissolving the digested samples in 7.5 mL of sodium citrate buffer (pH 2.2), filtration and centrifugation (2680 g for 15 min) were applied. Then, 1.0 μL of the prepared samples was injected into an RP‐HPLC Agilent 1100 (Agilent Technologies, Palo Alto, CA, USA) with a column Agilent Hypersil ODS (250 mm, 4.0 mm, 5 μm) at 40°C, with a coupled ultraviolet (UV) detector (Variable Wavelength Detectors, VWD) at 338 nm and 262 nm for proline. While the mobile phase flow rate was 1 mL/min, sodium acetate (27.6 mmol/L)/triethylamine/tetrahydrofuran (500:0.11:2.5, *v/v/v*) and sodium acetate (80.9 mmol/L)/methanol/acetonitrile (1:2:2, v/v/v) were the mobile phase A (pH 7.2) and the mobile phase B (pH 7.2) (Al‐Ansi et al. 2020).

### 2.8 Sensory Evaluation

Sensory evaluation of the bread samples was implemented on the day of production by 19 panelists (10 men and 9 women, ages between 24 and 40 years) with experience in the sensory evaluation of bread at the cereal research center at Tehran Medical Sciences, Islamic Azad University (Tehran, Iran). The bread samples were coded with three‐digit numbers and scored for color (scale 1–5, dark brown to creamy), flavor (Odor and taste, scale 1–5, unpleasant to pleasant), acidic taste (scale 1–5, over‐acidic to mild‐acidic), crumb texture (scale 1–5, hard to soft) and overall acceptance (scale 1–5, low to high) (Bernaert et al. 2022). The panelists used tap water before testing each sample.

### 2.9 Statistical Analysis

Three bread‐making trials were performed, and each analysis was done in two replicates. The effect of sourdough type on the quality and functional characteristics of bread samples was analyzed by One‐Way ANOVA using the SPSS program (SPSS package program, version 22.0, IBM Corp., NY, USA). Duncan's multiple range test was used for comparing the means of different treatments (significance level = 0.05).

## 3 Results and Discussion

### 3.1 Chemical Composition

The chemical compositions of bread samples are presented in Table 1. The results revealed a significant decrease (*p* ≤ 0.05) in the pH value of the sourdough breads in comparison with the control one, due to synthesis of higher levels of CO_2_ and organic acids, that is, lactic acid and acetic acid, by lactic acid bacteria during the fermentation process (Pinto, Oliveira, and Pintado 2022; Gänzle and Zheng 2023). On the other hand, the pH of *L. sanfranciscensis‐containing* bread was significantly lower than those of other sourdough breads. The reason might be related to the synthesis higher contents of lactic acid by *L. sanfranciscensis* compared with *L. sakei* (Harth, Kerrebroeck, and Vuyst 2018). Furthermore, higher sensorial quality characteristics of breads containing lactic acid bacteria with a higher acidification rate have been documented in several researches (Garzon et al. 2021; Gänzle and Zheng 2023; Tomić et al. 2023).

**TABLE 1:** The chemical composition of control and sourdough bread samples (*n* = 3).

Sample	A	B	C	D
pH	4.53 ± 0.02^a^	4.09 ± 0.02^b^	3.92 ± 0.01^c^	3.96 ± 0.01^c^
Moisture (%)	34.97 ± 0.10^d^	35.40 ± 0.10^b^	35.03 ± 0.11^c^	36.16 ± 0.13^a^
Protein (%)	9.65 ± 0.10^a^	8.92 ± 0.08^b^	7.30 ± 0.14^d^	8.41 ± 0.10^c^

*Note:* Data are shown as the mean ± SD of experiments that were conducted at least in triplicate. Different superscripts within the same raw indicate significant differences at *p* ≤ 0.05. A: control bread, B: bread prepared with sourdough containing *L. sakei* , C: bread prepared with sourdough containing *L. sanfranciscensis* , D: bread prepared with sourdough containing the mixture of *L. sakei* and *L. sanfranciscensis* .

According to the results, while the lowest moisture content was observed in the control bread, the bread prepared with sourdough containing the mixture of *L. sakei* and *L. sanfranciscensis* had the highest moisture value (*p* ≤ 0.05). The higher content of moisture in sourdough breads compared to control bread can be linked to the weakening the water holding capacity of the proteins due to the presence of acidic conditions created by fermentation (Longin et al. 2023).

The highest and lowest values of protein were observed in the control and *L. sanfranciscensis* fermented breads, respectively. Degradation of proteins during the fermentation process in sourdough breads can decrease the protein content in the sourdough breads. Moreover, hydrolysis of proteins and reducing the phytic acid content during the fermentation process of sourdough breads result in breads with more digestible proteins and higher mineral bioavailability compared to conventional ones (Olojede, Sanni, and Banwo 2020; Suo, Chen, and Wang 2021).

### 3.2 Free and Bound Phenolic Compounds

**FIGURE 1:**
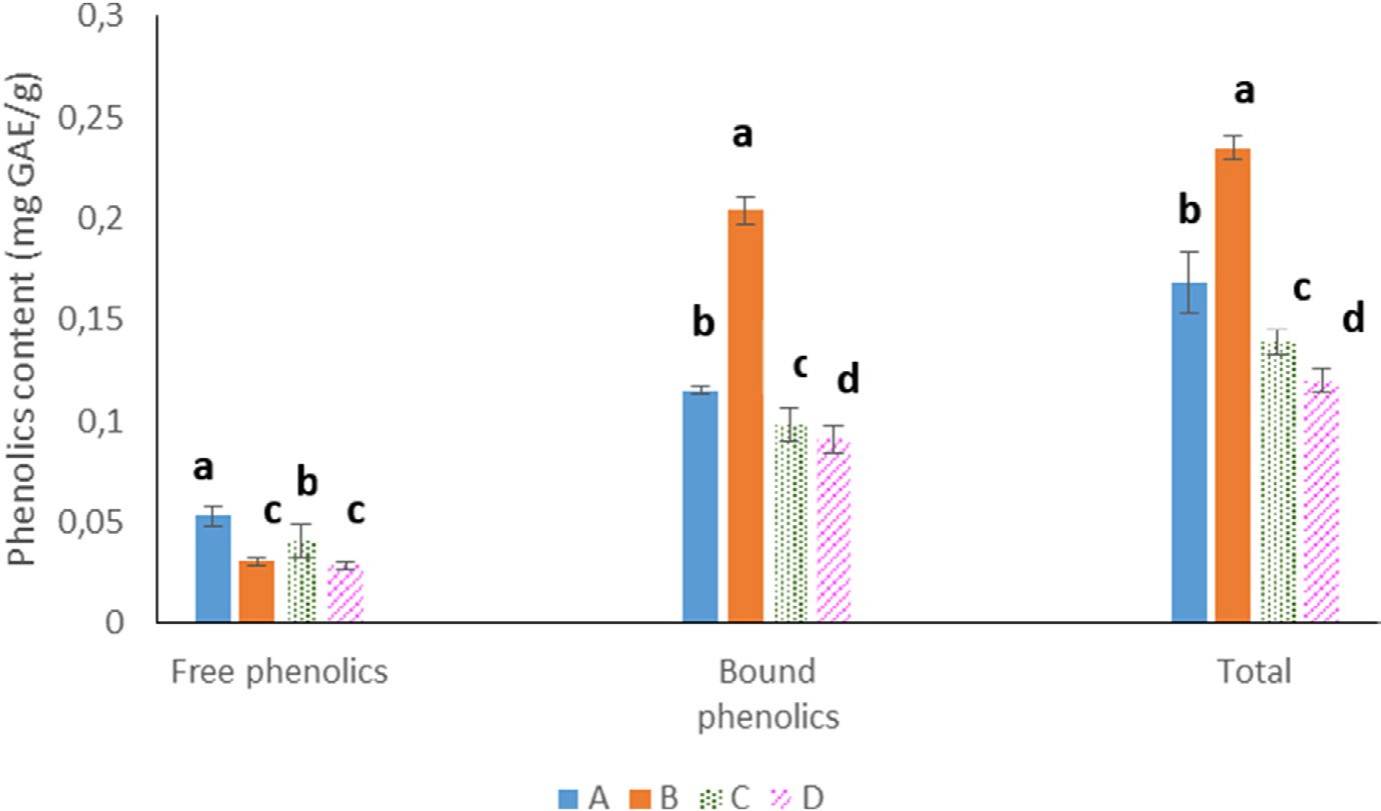
The amounts of free, bound, and total phenolic compounds of control and sourdough bread samples. (A) Control bread, (B) bread prepared with sourdough containing *L. sakei* , (C) bread prepared with sourdough containing *L. sanfranciscensis* , (D) bread prepared with sourdough containing the mixture of *L. sakei* and *L. sanfranciscensis* . Data are presented as mean ± SD. Different superscripts indicate significant differences at *p* ≤ 0.05.

Phenolic compounds as secondary metabolites in plants, owing to their antioxidant property, capability in lowering the glycemic index of foods, and antidiabetic effects, are considered health beneficial compounds (Ripari, Bai, and Gänzle 2019). In the current study, the content of bound phenolic compounds was by far higher than free phenolics in all samples. Control bread had the highest amount of free phenolic compounds, while the *L. sakei* fermented sourdough bread possessed the highest amounts of bound and total phenolics (Figure 1). The microbiota of the fermentation medium and their inherent enzymes are responsible for phenolic compounds metabolism. The increase of bound phenolics can be explained by the esterase activity of the microorganisms involved in sourdough fermentation (Ravisankar, Dizlek, and Awika 2021) and the dimerization/polymerization of free phenolic acids (Antognoni et al. 2019). Higher amounts of bound phenolics compared to free ones in fermented wheat have also been documented earlier (Anson et al. 2009; Antognoni et al. 2019). On the other hand, the low amount of bound phenolic compounds in the sourdough bread prepared with a mixture of *L. sakei* and *L. sanfranciscensis* , compared to other samples, could be attributed to the antimicrobial effect of sakacin against *L. sanfranciscensis*. Sakacins are a category of bacteriocins secreted by certain strains of *L. sakei* and are active against *Listeria spp*. and different strains of *Lactobacillus spp*. (Delves‐Broughton 2012). It seems that the probable presence of sakacins during the co‐fermentation of sourdough by *L. sakei* and *L. sanfranciscensis* retards the activity of *L. sanfranciscensis* and its inherent enzymes involved in phenolics metabolism, and as a result, the release of bound phenolics is diminished.

Following the changes of free phenolic compounds (Figure 1) showed the highest amount of these compounds in control and the sample fermented with *L. sanfranciscensis*. The concentrations of total phenolic compounds in sourdough breads prepared with *L. sakei* and a mixture of *L. sakei* and *L. sanfranciscensis* were the highest and the lowest among samples, respectively, which was expectable because the total phenolic content parameter has been reported as the sum of the content of free and bound phenolic components. In general, studying the variations in the concentration of free and bound phenolic compounds indicates different behavior of the bacterial strains used in the metabolism of phenolic compounds. Bacterial strains have different behaviors in degrading tannins or phenolic acid esters by their esterase and phenolic acid decarboxylase enzymes (Antognoni et al. 2019).

### 3.3 Antioxidant Capacity

**FIGURE 2:**
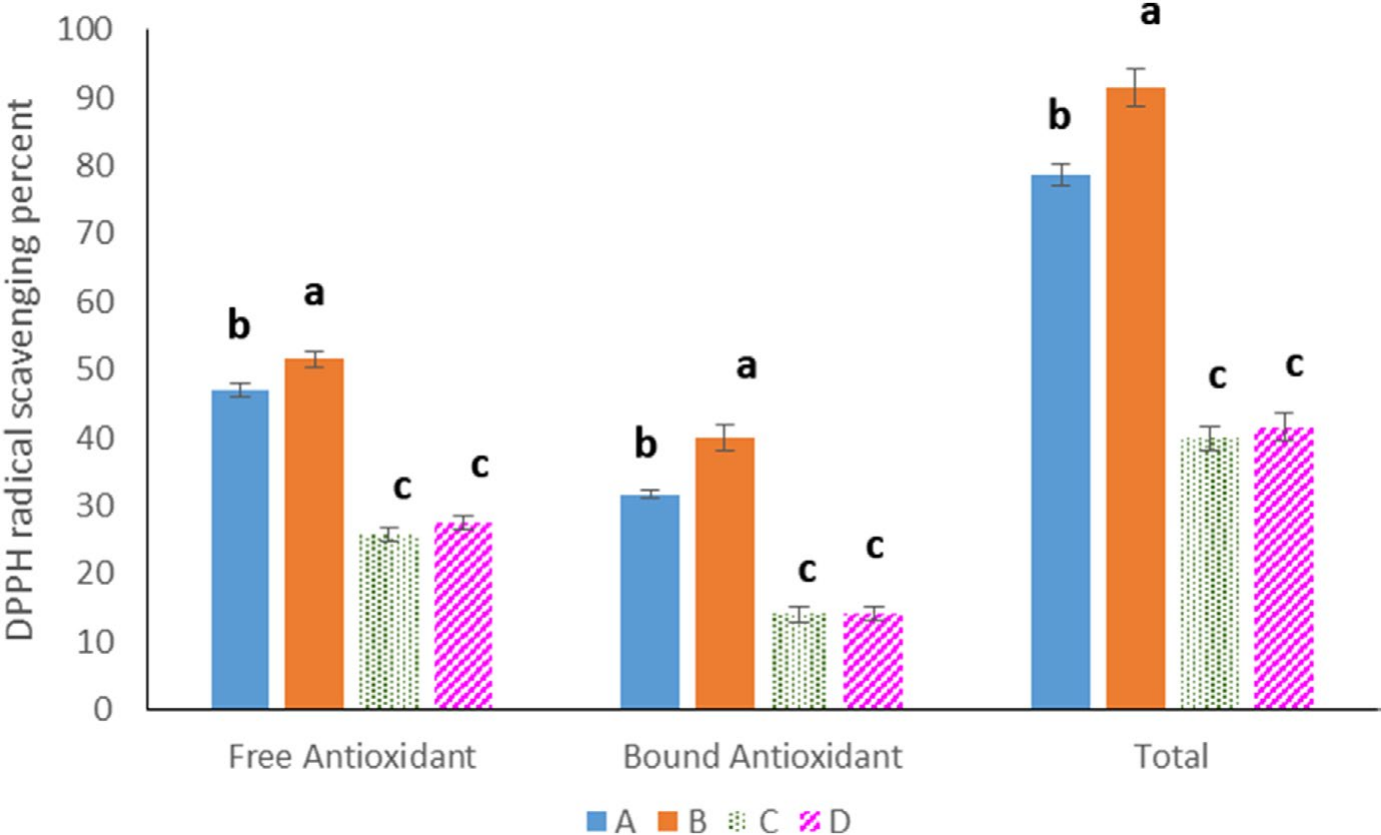
The antioxidant activity of free, bound, and total phenolic compounds in control and sourdough bread samples. (A) Control Bread, (B) bread prepared with sourdough containing *L. sakei* , (C) bread prepared with sourdough containing *L. sanfranciscensis* , (D) bread prepared with sourdough containing the mixture of *L. sakei* and *L. sanfranciscensis* . Data are presented as mean ± SD. Different superscripts indicate significant differences at *p* ≤ 0.05.

The bioactive components of cereals have been the focus of research in recent years. Wheat flour, as the main constituent of bakery products, contains nutrients (e.g., protein, starch, dietary fibers, minerals) and bioactive substances such as phenolic compounds (Li et al. 2024). Many of the phenolic compounds play an antioxidant role through scavenging free radicals, sequestering pro‐oxidant metal ions, and neutralizing reactive oxygen species (Yang et al. 2024). On the other hand, sourdough fermentation is a tool for subjecting flour constituents to substrates of enzymes and bacterial strains and the synthesis of bioactive (such as antioxidants) or nutritious metabolites (Antognoni et al. 2019). In the current study, the preparation of sourdoughs with *L. sakei* resulted in breads with the highest total antioxidant capacity, while fermentation of sourdoughs with *L. sanfranciscensis* or the co‐culture of *L. sakei* + *L. sanfranciscensis* led to the production of breads with the lowest total antioxidant capacities (Figure 2). Also, the comparison of the antioxidant power of free and bound phenolic compounds in each treatment indicated the higher antioxidant activity of free phenolic compounds than the bound types. The reason could be attributed to the chemistry and structure of phenolic compounds. The architecture of these compounds consists of an aromatic ring with one or more hydroxyl substituents. They are generally classified as phenolic acids, flavonoids, and tannins. In cereal grains, phenolic acids, as the simplest form of phenolic compounds, are mostly esterified to arabinoxylans and make bridges between hemicellulose molecules (Duodu 2011). It seems that the binding of phenolics with different molecules, such as carbohydrates and proteins, restricts the ability of phenolic compounds to act as a strong antioxidant. In general, the results of this study indicate that the antioxidant power of free and bound phenolic compounds of sourdough breads is related to the type of the microbial strain used for fermenting sourdough and the activity of endogenous and bacterial enzymes in the release of free and soluble bound phenolic compounds with radical scavenging activity.

### 3.4 GABA Content

GABA is a four‐carbon nonprotein amino acid that is found in many plant and animal cells. Along with beneficial effects for the human nervous system, GABA has important health‐related roles, that is, anti‐inflammatory, antidiabetes, and antihypertensive properties. Considering the remarkable biological activities of GABA, the food industry pays great attention to the production of GABA‐enriched foods (Diez‐Gutiérrez et al. 2020; Hou et al. 2023). Although GABA can be synthesized by the plants, microorganisms and potentially lactic acid bacteria are more promising candidates for its production because of faster growth rates, cultivation under controlled conditions, and no need for large cultivation areas (Cui et al. 2020; Kittibunchakul et al. 2021).

**FIGURE 3:**
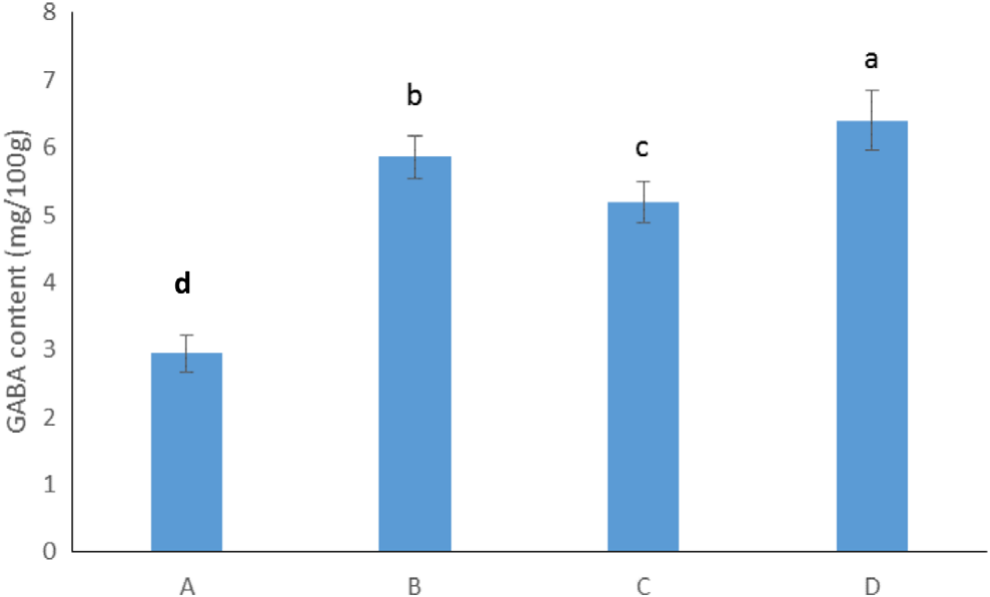
The GABA content in control and sourdough bread samples. (A) Control bread, (B) bread prepared with sourdough containing *L. sakei* , (C) bread prepared with sourdough containing *L. sanfranciscensis* , (D) bread prepared with sourdough containing the mixture of *L. sakei* and *L. sanfranciscensis* . Data are presented as mean ± SD. Different superscripts indicate significant differences at *p* ≤ 0.05.

The GABA contents of bread samples are shown in Figure 3. Overall, sourdough fermentation caused an increase in GABA content in the bread samples. The breads prepared with different sourdoughs had higher GABA content than the control one. Fermentation of sourdough by lactic acid bacteria has been reported as a key parameter for the synthesis of GABA in sourdough breads (Diana, Rafecas, and Quílez 2014; Xuan et al. 2024).

On the other hand, significant differences were seen among the sourdough breads prepared using different lactic acid bacteria (*p* ≤ 0.05). In this context, using *L. sakei* for sourdough fermentation led to the synthesis of higher GABA content in the final bread compared with *L. sanfranciscensis* . The highest GABA content was observed in the bread prepared with a mixture of *L. sakei* and *L. sanfranciscensis* . This difference can be linked to the different GABA producing capability of the lactic acid bacteria used for sourdough fermentation, where the conversion of glutamic acid to GABA is dependent on the catalytic activity of the decarboxylase enzyme of the specific strain used (Venturi et al. 2019; Xuan et al. 2024). According to the results, it can be stated that co‐fermentation of sourdough by a mixture of *L. sakei* and *L. sanfranciscensis* results in the synthesis of higher contents of GABA compared to the use of a single strain (*p* ≤ 0.05).

### 3.5 Free and Total Amino Acid Content

The amino acid composition of bread samples is presented in Table 2. As shown, the sample fermented with a mixture of *L. sakei* and *L. sanfranciscensis* had the highest amount of essential, nonessential, and total free amino acids among the sourdough breads, which indicates that the dough is more proteolyzed in this bread. Fermentation can increase the rate of protein hydrolysis via lactic acid bacteria and microbial enzymes, lead to the breakdown of proteins to amino acids, and increase the level of free amino acids in bread (Koistinen et al. 2018; Limbad et al. 2020).

**TABLE 2:** The amino acid profile of control and sourdough bread samples (*n* = 3).

Amino acids (mg/100 g)	A	B	C	D
Essential amino acids				
Lysine	147.21 ± 3.77^a^	132.46 ± 4.41^c^	139.61 ± 3.88^b^	138.97 ± 5.12^b^
Lucine	422.02 ± 7.52^a^	432.13 ± 5.39^a^	285.71 ± 4.66^b^	424.53 ± 5.80^a^
Isoleucine	267.17 ± 8.34^a^	237.87 ± 5.77^c^	246.75 ± 6.69^b^	204.12 ± 4.68^d^
Histidine	151.58 ± 6.18^a^	119.43 ± 4.25^d^	142.85 ± 4.56^b^	132.46 ± 3.80^c^
Arginine	327.15 ± 8.75^c^	412.59 ± 7.93^a^	418.83 ± 8.18^a^	398.47 ± 8.05^b^
Valin	368.59 ± 10.18^a^	238.87 ± 6.69^d^	357.14 ± 8.33^b^	338.76 ± 4.56c
Threonine	161.39 ± 3.90^d^	187.83 ± 4.07^c^	214.28 ± 6.11^a^	203.04 ± 6.28^b^
Methionine	162.48 ± 4.15^b^	120.52 ± 3.55^d^	177.48 ± 3.72^a^	140.06 ± 4.21^c^
Phenylalanine	249.72 ± 5.58^b^	250.81 ± 4.47^b^	251.08 ± 4.65^b^	269.27 ± 5.70^a^
Total EAA	2257.31 ± 58.37^a^	2132.51 ± 46.53^d^	2233.73 ± 50.78^c^	2249.68 ± 48.20^b^
Nonessential amino acids				
Glutamic acid	415.92 ± 6.35^d^	466.88 ± 7.85^c^	507.57 ± 6.47^b^	715.52 ± 8.24^a^
Glycine	213.74 ± 4.65^a^	200.86 ± 4.10^b^	211.03 ± 6.72^a^	203.04 ± 4.85^b^
Alanine	316.24 ± 9.66^b^	358.30 ± 8.15^a^	307.35 ± 4.55^b^	366.99 ± 7.33^a^
Cysteine	47.98 ± 3.24^c^	52.11 ± 3.47^bc^	89.82 ± 5.22^a^	56.46 ± 6.18^b^
Aspartic acid	482.01 ± 13.56^a^	456.02 ± 15.71^c^	474.02 ± 15.55^b^	484.25 ± 14.32^a^
Serine	296.61 ± 9.58^c^	371.33 ± 11.68^a^	308.44 ± 11.27^b^	311.61 ± 6.80^b^
Tyrosine	285.71 ± 6.55^b^	281.21 ± 8.90^b^	305.19 ± 11.47^a^	274.70 ± 7.70^c^
Tryptophan	52.34 ± 4.55^c^	82.51 ± 7.82^a^	46.53 ± 6.11^d^	58.63 ± 5.43^b^
Proline	209.37 ± 9.45^a^	208.46 ± 5.69^a^	194.80 ± 8.85^b^	184.58 ± 7.57^c^
Total NEAA	2119.92 ± 67.59^c^	2477.68 ± 73.37^b^	2444.75 ± 76.21^b^	2655.78 ± 68.42^a^
Total AA	4377.23 ± 125.96^d^	4610.19 ± 119.90^c^	4678.48 ± 126.99^b^	4905.46 ± 116.62^a^

*Note:* Data are shown as the mean ± SD of experiments that were conducted at least in triplicate. Different superscripts within the same raw indicate significant differences at *P* ≤ 0.05. A: Control Bread, B: bread prepared with sourdough containing *L. sakei* , C: bread prepared with sourdough containing *L. sanfranciscensis* , D: bread prepared with sourdough containing the mixture of *L. sakei* and *L. sanfranciscensis* .

Abbreviations: AA, amino acids; EAA, essential amino acids; NEAA, nonessential amino acids.

*L. sakei* + *L. sanfranciscensis* fermented bread also showed the highest amount of essential amino acids among the sourdough breads, which is important from a nutritional point of view, since essential amino acids cannot be synthesized by humans and must be supplied from a diet. In the current study, the most concentrated amino acids in all sourdough breads were glutamic acid and aspartic acid. The highest content of these two mentioned amino acids was observed in sourdough bread prepared with a mixture of *L. sakei* and *L. sanfranciscensis* .

### 3.6 Sensory Evaluation

**FIGURE 4:**
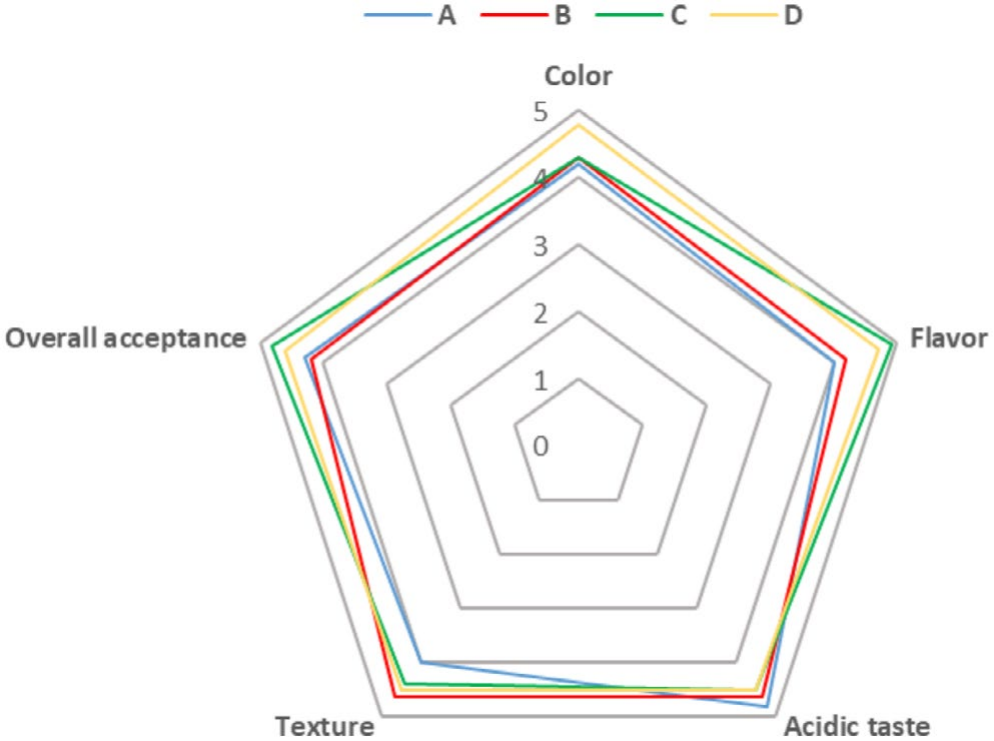
The sensory characteristics of control and sourdough bread samples: (A) Control bread, (B) bread prepared with sourdough containing *L. sakei* , (C) bread prepared with sourdough containing *L. sanfranciscensis* , (D) bread prepared with sourdough containing the mixture of *L. sakei* and *L. sanfranciscensis* .

The sensory characteristics of bread samples are presented in Figure 4. Evaluation of the color of breads showed a significant difference between the sourdough bread containing both *L. sakei* and *L. sanfranciscensis* with other samples (*p* ≤ 0.05). The mentioned sample had a more pleasant golden brown color from the panelists’ point of view. The color of bread is influenced by caramelization and Maillard reactions. The Maillard reaction between amino nitrogen and reducing sugars causes a light to gold‐brown color in bread. The use of microorganisms with high proteolytic power for the fermentation of bread dough causes more hydrolysis of proteins and the release of more free amino acids in the dough medium. Furthermore, a pH drop occurs due to the production of organic acids. All the mentioned conditions favor the occurrence of the Maillard reaction and the formation of light to dark brown pigments during the baking of bread (Seis Subaşı and Ercan 2024). In the present research, the highest content of free amino acids (Table 2) and the lowest pH (Table 1) were observed at sample D, which was co‐fermented by *L. sakei* and *L. sanfranciscensis*. These observations confirm the effect of pH and the amount of free amino acids on the occurrence of the Maillard reaction and the color of the sample.

Since odor and taste are important sensorial characteristics of a food product and have a direct effect on choosing a product by consumers, in this study the flavor (as an indicator of odor and taste) and also the probable sensation of acidic taste were evaluated in bread samples. As shown in Figure 4, sourdough breads containing *L. sanfranciscensis* (sample C) and *L. sakei* + *L. sanfranciscensis* (sample D) had almost similar flavor scores (*p* > 0.05), while their difference was significant with the flavor of control and *L. sakei* containing samples (*p* ≤ 0.05). Acidic taste is an important sensorial parameter in sourdough breads. Synthesis of different organic acids such as lactic and acetic acid by lactic acid bacteria during sourdough fermentation can lead to the formation of sour taste in the final product (Cera et al. 2024; Wang and Wang 2024). However, using appropriate bacteria and well‐controlled sourdough fermentation can result in the preparation of bread with desirable taste and flavor (Cera et al. 2024). In this study, an unpleasant acidic taste was not reported in any of the breads.

In evaluating the texture of the samples (hardness and softness), the sourdough breads possessed a softer and more pleasant texture than the control sample (Figure 4). Synthesis of organic acids by lactic acid bacteria and subsequent pH drop increase the solubility of proteins and activity of proteolytic enzymes, which leads to the softening of bread texture and improvement of dough rheology (Zahra et al. 2022; Zhang et al. 2023). In general, the highest score for overall acceptance of the breads was assigned to C and D sourdough breads (*p* > 0.05).

## 4 Conclusion

A new sourdough bread was developed by using *L. sakei* and germinated brown rice. For this purpose, three types of sourdoughs consisting of *L. sakei, L. sanfranciscensis*, and a mixture of *L. sakei* + *L. sanfranciscensis* were formulated. Germinated brown rice flour was used as the main ingredient of sourdoughs, and baker's yeast was added to all sourdough samples. Each sourdough was used for the preparation of a bread. Evaluation of the functional and quality characteristics of the breads indicated a higher content of phenolic compounds and antioxidant activity of *L. sakei* sourdough bread compared with the control and other bread samples. The highest GABA content was observed in *L. sakei* + *L. sanfranciscensis* sourdough bread, followed by *L. sakei* fermented bread. The highest overall acceptability was assigned to *L. sakei* + *L. sanfranciscensis* and *L. sanfranciscensis* sourdough bread samples. As a conclusion, *L. sakei* has the potential to be used in the functional bakery industry. Studying the other functional features of this food‐grade microorganism in fermented bakery products is recommended.

## Ethics Statement

This study does not involve any human or animal testing.

## Consent

Written informed consent was obtained from all study participants.

## Conflicts of Interest

The authors declare no conflicts of interest.

## Data Availability

The data that support the findings of this study are available from the corresponding author upon reasonable request.
